# A Comparison between Kidney Allograft Biopsies Performed by Nephrologists and Surgeons Versus Interventional Radiologists

**DOI:** 10.7759/cureus.6315

**Published:** 2019-12-06

**Authors:** Sandeep Aggarwal, Waqas J Siddiqui, Nauman Shahid, Jaime Baynes, Muhammad W Khattak, Irfan Ahmed, Suganthi Soundararajan, Ziauddin Ahmed

**Affiliations:** 2 Cardiology/Nephrology, Drexel University College of Medicine, Philadelphia, USA; 3 Nephrology, Eastern Nephrology Associates, Greenville, USA; 4 Nephrology, Hahnemann University Hospital, Drexel University, Philadelphia, USA; 5 Nephrology, Unity Point Health Methodist, Illinois Kidney Disease and Hypertension Center, Peoria, USA; 6 Nephrology, Arizona Kidney Disease & Hypertension Center, Chandler, USA; 7 Pathology, Hahnemann University Hospital, Drexel University, Philadelphia, USA

**Keywords:** clinical nephrology, interventional radiology, surgery, renal biopsy

## Abstract

Introduction: Renal biopsy is an integral part of clinical nephrology practice that helps in the diagnosis of various renal diseases. Across the globe, it is performed by nephrologists and/or surgeons under ultrasound guidance. Lately, this novel procedure has been performed more frequently by the interventional radiologist (IR) as compared to nephrologists and surgeons.

Methods: We completed a retrospective review of 378 consecutive renal biopsies performed at our university hospital in the city center of Philadelphia, Pennsylvania, between September 2008 and June 2011 for various indications. Baseline characteristics were comparable except systolic blood pressure (SBP), prothrombin time (PT), and international normalized ratio (INR) which was higher. Hemoglobin was lower in patients who underwent biopsy by the IR compared to those who were biopsied by nephrologists and/or surgeons.

Results: The primary outcome showed the average number of glomeruli obtained with each biopsy was significantly lower by nephrology or surgical teams, 9.09 ± 5.17 vs. 19.17 ± 11.11 obtained by the interventional radiology team, p-value <0.0001. The number of cores obtained with each biopsy was significantly lower by nephrologist or surgeon at the bedside, 1.57 ± 1.05 vs. 2.42 ± 1.26, p-value <0.0001. The average number of attempts to obtain one core was 2.00 ± 1.10 vs. 2.60 ± 1.17 by nephrologist and surgeon vs. IR, respectively, p-value <0.0001.

Conclusion: Our study clearly shows the superior success of renal biopsy by the IR as compared to the nephrology and surgical teams. This calls for more robust training of nephrology fellows and surgery residents to obtain the renal biopsy to prevent the loss of this unique procedure skill by non-radiology clinicians.

## Introduction

Kidney biopsies are indicated to make the diagnosis in patients with various renal diseases, including acute or chronic renal failure, delayed graft function, unexplained hematuria, and proteinuria [1-3]. Several complications have been associated with the procedure, such as perinephric hematoma, gross hematuria, hospitalization, arterio-venous fistula (AVF), and bowel perforations [3-4]. Most of the renal procedures, including renal biopsies, are part of the core curriculum of the Nephrology fellowship all over the world [5-6] and in most centers around the United States (US) [5-7]. Recently, renal biopsies are increasingly being performed by interventional radiologists (IR) compared to the nephrologists in training and surgeons who use the bedside ultrasound-guided percutaneous technique [8-10]. This is due to the loss of follow-up care and lack of training skills by the nephrology fellows as compared to the IR [10]. This transition has occurred to limit the complications associated with the ultrasound-guided bedside technique and to increase the safety, diagnostic yield, and efficacy of the procedure.

The number of glomeruli and the average number of cores obtained during the allograft biopsy is a cornerstone to increase the yield of the diagnostic specimen [10-11]. The number of attempts that it takes to obtain the precise sample is also essential because it decreases the chance of complications associated with the procedure [10-11]. There have been limited studies done to date comparing the efficacy, safety, and yield of renal biopsy and the cost-effectiveness between the bedside technique performed by the nephrology fellows and surgeons compared to the biopsy performed by the interventional radiologist [10]. Thus to compare both techniques, we performed this retrospective analysis to elucidate the factual difference that exists between the two methods and to identify if there is any significant superiority that lies between the two techniques.

## Materials and methods

We completed a retrospective review of 378 consecutive renal biopsies done at our university hospital in the city center of Philadelphia, Pennsylvania between September 2008 and June 2011. Three hundred and seventy-eight biopsies were performed for various indications including protocol biopsies, rejection, and delayed graft function (DGF) or other reasons. Two hundred and forty biopsies were performed at the bedside after attaining all aseptic measures under the grayscale ultrasound guidance without biopsy needle guide, either by the nephrology fellows, nephrology attendings, the surgical residents and surgery attendings. One hundred and thirty-eight biopsies were done by the IR team in the radiology department. Eighteen gauge needles were used for all biopsies. The baseline characteristics of the patients who underwent renal biopsies are summarized in Table 1.

**Table 1:** Baseline Characteristics n. = Number, SBP = Systolic Blood Pressure, DBP = Diastolic Blood Pressure, MAP = Mean Arterial Pressure, PT = Prothrombin Time, INR = International Normalized Ratio, PTT = Partial Thromboplastin Time.

	Bed Side	Interventional Radiology Suite	p-value
n.	240	138	
Age	53.11 ± 13.5	52.4 ± 13.13	0.816
Males/Females	156/84	94/44	0.537
African American	134	82	0.5
SBP	135.84 ± 17.86	143.91 ± 24.73	0.0165
DBP	75.69 ± 13.58	76.18 ± 15.99	0.826
MAP	93.11 ± 23.8	81.68 ± 40.42	0.053
Hemoglobin	12.00 ± 1.83	10.55 ± 2.02	0.001
Platelets	217.51 ± 71.24	211.45 ± 74.77	0.546
PT	11.79 ± 1.35	12.36 ± 1.58	0.007
INR	1.03 ± 0.11	1.07 ± 0.13	0.01
PTT	32.10 ± 4.60	33.00 ± 10.08	0.466

Age, African American race, and gender were equally matched. Systolic blood pressure was significantly higher in the patients who underwent biopsy by the IR but there was no difference in the diastolic blood pressure or the mean arterial pressure of the patients. Prothrombin time (PT) and international normalized ratio (INR) was also significantly higher in patients who got the biopsy with IR compared to the biopsies done at the bedside. There was significantly lower hemoglobin (Hgb) among patients who underwent the biopsy in the radiology department compared to the patients who got the biopsy at the bedside. There was no difference in partial thromboplastin time (PTT) or the platelet count as seen in Table 1.

Primary outcome

The primary outcomes that were assessed were 1) the average number of glomerulus obtained with each biopsy, 2) average number of cores obtained with each biopsy and 3) average number of attempts to obtain one core.

Secondary outcome

The secondary outcomes were mainly the anticipated complications of a renal biopsy and they were 1) the procedural complication of gross hematuria, 2) need of hospitalization for any reason after the procedure, 3) perinephric hematoma, 4) AVF formation, 5) bowel perforation, 6) need for surgical intervention, and 7) need for blood transfusion.

Statistical method

IBM Statistical Package for the Social Sciences (SPSS Inc., Chicago, IL) was used to calculate the statistical significance by comparing the means of the individual outcome. Chi-square test was used and the value of < 0.05 was considered to be statistically significant.

## Results

The primary outcome included the average number of glomerulus obtained with each biopsy was 9.09 ± 5.17 by the nephrologist or the surgical team in the bedside group compared to 19.17 ± 11.11 obtained by the IR in the radiology department, p-value <0.0001. The number of cores obtained with each biopsy at the bedside or by IR was 1.57 ± 1.05 vs. 2.42 ± 1.26, respectively p-value <0.0001. The average number of attempts to obtain one core was 2.00 ± 1.10 vs. 2.60 ± 1.17 at the bedside and IR, respectively, p-value <0.0001 (Table 2).

**Table 2:** Primary Outcomes - Allograft Biopsy Tissue Yield p-value calculated using chi-square test.

	Biopsy by Nephrologist or Surgeon at bed-side	Biopsy by Interventional Radiologist	p-value
n.	240	138	
Numbers of Glomerulus	9.09 ± 5.17	19.17 ± 11.11	<0.0001
Numbers of Crores	1.57 ± 1.05	2.42 ± 1.26	<0.0001
Attempts per core	2.00 ± 1.10	2.60 ± 1.17	<0.0001

The secondary outcomes showed that the most common complications were gross hematuria in four cases in the bedside group vs. one case in the IR group, p-value = 0.4402. Patients requiring hospitalizations for the complications of the biopsy were six in the bedside biopsy group vs. one in the IR group, p-value = 0.2177. One case of perinephric hematoma was observed in each group, p-value = 0.691. There were two cases of the arteriovenous fistula in the bedside group but none in the IR group. There were no events of bowel perforation, need for surgical intervention, or need for blood transfusion in either group (Table 3).

**Table 3:** Secondary Outcomes - Complications p-value calculated using chi-square test.

	Biopsy by Nephrologist or Surgeon at bed side	Biopsy by Interventional Radiologist	p-value
n.	240	138	
Gross Hematuria n. (%)	4 (1.66)	1 (0.72)	0.4402
Hospitalization n. (%)	6 (2.5)	1 (0.72)	0.2177
Perinephric Hematoma n.	1 (0.41)	1 (0.72)	0.691
Arterio-Venous Fistula n. (%)	2 (0.83)	0	
Bowel Perforation	None	None	
Need for surgical intervention n. (%)	None	None	
Blood Transfusion n.	None	None	

## Discussion

Although this study shows a fewer number of cores and fewer attempts per core with bedside biopsies, it should be noticed that this was a single-center study with limited sample size. Moreover, the sample size was not equally distributed, with a significantly higher number of patients in the bedside group. It should also be noted that systolic blood pressure (SBP) and INR were, on average, higher in the IR biopsy group, which theoretically predispose those patients to higher post-procedural complications. Hence, having relatively fewer IR related biopsy complications, as compared to bedside biopsies, points towards better techniques by experienced interventional radiologists.

This study provides a platform and lays a foundation for future studies by other academic institutions where patient’s safety, cost-effectiveness, and training the residents and fellows are valued and evaluated. The number of glomeruli and the average number of cores obtained during a procedure is considered a cornerstone to increasing the yield of the diagnostic specimen [10-11]. The number of attempts that it takes to obtain the precise specimen is also important because it decreases the chance of complications associated with the procedure [10-11]. There have been limited studies done to date to compare the efficacy, safety, and yield of renal biopsy along with the cost-effectiveness between the bedside technique. To implement the routine bedside biopsies safely and effectively, an institution like ours need to devote resources for hands-on fellows and faculty training. This can be achieved by a dedicated period of training for residents and fellows during their training to learn, practice, and be proficient in these procedures.

In summary, the bedside biopsies, if done in a correct patient population, can be safe, efficient, and cost-effective. It can be a major component of fellow’s or residents' training as well as a key factor in reducing the healthcare financial burden on society.

## Conclusions

Renal biopsies have been part of the nephrology and surgical practice. With the recent advent of interventional techniques, more and more renal biopsies are being performed by the IRs. Thus making the future nephrologists less comfortable with bedside biopsies leading to an increased number of biopsies being performed by the IR. This leads to an increase in healthcare costs. A structured and dedicated time as part of the nephrology fellowship and surgical training will aide in providing skills for a safe and successful biopsy at the bedside. It can, in turn, minimize the interventional radiology referrals, healthcare costs, and delay patient care.
